# Ushering in a new era of RNA-based therapies

**DOI:** 10.1038/s42003-021-02150-w

**Published:** 2021-05-17

**Authors:** Robert DeLong

**Affiliations:** College of Veterinary Medicine, Kansas State University, Manhattan, KS USA

## Abstract

A recent meeting at Cold Spring Harbor Laboratory focused on emerging nucleic acid therapies and the essential academic research that has enabled them. The program encompassed everything from chemical innovations to preclinical and clinical progress, and provided a glimpse of the breakthroughs yet to come.

With the rapid approval of the mRNA-based vaccine for emergency use in the fight against COVID-19 there is an increased public awareness in the potential of RNA-based medicines. Thus the recent Cold Spring Harbor Laboratory meeting on Nucleic Acid Therapies convening many of the world’s leaders could not have been more timely, or exciting.

Beyond COVID-19, the delivery of mRNA vaccines against other diseases, including cancer, seems now poised to achieve great success.

The importance of fundamental advances in chemistry and biology to development of cutting-edge pharmaceuticals was a key theme of the meeting. RNA being perhaps one of the least biologically stable of the macromolecules, it is uniquely versatile in its application to novel therapies. RNA can be modified in the base, backbone and sugar to increase target affinity, to thwart nuclease digestion and to maintain the drug’s chemical integrity once administered in vivo. Shulin Gao at Ionis Pharmaceuticals provided an overview of the progress made in RNA modifications and their impact on drug development since the late 1980s, when first generation chemistries—phosphorothioates (PS) modified with a sulfur on their phosphate backbone—were developed. Further innovations on this theme have led to significant improvements in therapies. For example, replacing the 2′-hydroxyl group on the ribose sugar of the PS backbone with a methoxyethoxy group improves target affinity and hence therapeutic activity. A talk from Muthiah (Mano) Manoharan at Alnylam Pharmaceuticals highlighted more recent developments, such as LICA (GalNac conjugates) that improve tissue uptake and biodistribution, showing particular promise against liver diseases.

An iconic example of fundamental research in RNA biology driving innovation in therapeutics is the development of CRISPR-Cas gene editing. Although already of great interest to researchers and pharmaceutical companies alike, the recent Nobel Prize in Chemistry awarded to Jennifer Doudna and Emmanuelle Charpentier for CRISPR technology has accelerated both research and drug development. Among the many highlights were three talks illustrating different avenues of current research. Samuel Sternberg at Columbia University presented a talk entitled, “Targeted DNA integration without double-strand breaks using CRISPR RNA-guided transposons,” describing a bacterially derived system—essentially an RNA-guided integrase enabling large-scale sequence changes without requiring double-strand DNA breaks and homologous recombination. Keith Gagnon and his group at Southern Illinois University focused on structure–function relationships in CRISPR-Cas12 and how varying the chemistry of the sgRNA pseudoknot affects the activity of this system. And finally, Briana Wilson at the University of Virginia demonstrated how blocking a specific cellular RNase stabilizes ultra-fast decaying small RNAs, thus promoting entry of small RNAs into the RNA-induced silencing complex (or RISC) as well as target repression. From the pharmaceuticals industry perspective, Christian Dombrowski and Kate Zhang from Intellia Therapeutics and Editas Medicine, respectively, provided insight into how companies are harnessing these discoveries. For example, cytosine base editing screening and optimization or engineering of AsCas12a to achieve high level on-target editing in hematopoietic stem cells, induced pluripotent stem cells, and natural killer (NK) cells, with promising results against sickle cell disease and antitumor activity.

Fittingly, the program ended with exciting preclinical and clinical progress. Anastasia Khvorova at the University of Massachusetts Medical School gave a beautiful talk demonstrating the efficacy of RNAi targeting against Alzheimer’s disease, achieving results in the brains of experimental mice where the plaques associated with this disease were clearly abrogated. We also heard from Arthur A. Levin at Avidity Biosciences Inc., who in his talk entitled, “Antibody oligonucleotide conjugates—Progressing towards the clinic”, showed that by conjugating ASO to antibody, the pharmacokinetics in monkeys could be greatly improved with pronounced increase in tissue distribution and prolonged gene silencing activity in multiple tissues. And lest we forget about the most important recent development in RNA-based therapies, Philip Dormitzer from Pfizer gave the keynote fireside chat entitled “COVID-19 Vaccine Development”.

Of course, a limitation to RNA-based therapies is that the targets are invariably within cells. Thus, much of the meeting focused on how to engineer RNA drugs that can overcome two main barriers to reaching its intended target within the cell. First, the RNA must be able to penetrate the cell membrane. On this point, Cosmin Mihai at Moderna, Inc. presented an elegant talk entitled, “Dissecting cytosolic mRNA delivery with single molecule resolution enables rational LNP design”, highlighting methods he developed to quantify mRNA in cells in order to evaluate lipids within the lipid nanoparticle (LNP) that might release more free RNA into the cytosol. Once inside the cell, the RNA needs to escape endosomal entrapment. Rudy Juliano—formerly at the University of North Carolina Chapel Hill—has now formed a company in the North Carolina Research Triangle Park called Initos Pharmaceuticals focused on exactly this problem. They aim to produce small molecular compounds that can be co-delivered with therapeutic nucleic acids and assist antisense and splice switching oligomers in endosomal escape.

The astounding speed with which vaccines were produced to battle COVID-19 has no doubt re-energized the community of researchers working on RNA biology and therapies. The promise of this field lies in combining all of the technologies discussed above and knowledge yet to be uncovered to develop novel therapies. Beyond COVID-19, the delivery of mRNA vaccines against other diseases, including cancer, seems now poised to achieve great success. We hope that in the aftermath of the pandemic, research effort (and funding) for both basic research and clinical applications of RNA biology will continue to advance with the aim of tackling traditionally intractable or rare diseases for which patients at the moment have little hope.

